# LY294002 may overcome 5-FU resistance via down-regulation of activated p-AKT in Epstein-Barr virus-positive gastric cancer cells

**DOI:** 10.1186/1471-2407-10-425

**Published:** 2010-08-13

**Authors:** Jung-Young Shin, Jeong-Oh Kim, Suk Kyeong Lee, Hiun-Suk Chae, Jin-Hyoung Kang

**Affiliations:** 1 Department of Biomedical Sciences, The Catholic University of Korea, Seoul, Korea; 2 Research Institute of Immunobiology, The Catholic University of Korea, Seoul, Korea; 3 Uijeongbu St. Mary's Hospital, The Catholic University of Korea, Gyeonggi-do, Korea; 4 Seoul St. Mary's Hospital, The Catholic University of Korea, Seoul, Korea

## Abstract

**Background:**

As EBV-associated gastric cancer has unique features that are different from EBV (-) gastric cancer, EBV is considered to have a key role in gastric carcinogenesis. It has been reported that viral latent membrane protein 2A (LMP2A) in EBV-transformed tumor cells activates the phosphatidylinositol 3-kinase (PI3K)/AKT pathway, which provides a survival signal and chemo-resistance to cytotoxic anti-cancer drugs. This study was to evaluate anti-proliferative effect and cell cycle change when 5-FU and LY294002 (LY), a selective inhibitor of PI3K, were treated separately or combined with different schedules in EBV positive gastric cancer cell line, SNU-719.

**Methods:**

After single treatment and sequential combination of 5-FU and LY, cytotoxic activity was measured by MTS assay. When 5-FU and LY were treated in single and sequential combinations, the expression of p-AKT, p-NFkB, p-p53 and bcl-2 was observed on different concentrations by Western blot analysis. We also investigated the effect on apoptosis and cell cycle distribution using flow cytometry. The LMP2A siRNA inhibition was done to confirm the reversal of decreased 5-FU activity and p-AKT.

**Results:**

When 5-FU was sequentially combined with LY, the combination index (CI) value indicated synergistic anti-proliferative effect. The expression of p-AKT and p-NFκB was upregulated by 5-FU alone but sequential treatment of 5-FU and LY decreased the expression of both p-AKT and p-NFκB. When 5-FU was combined with LY, G0/G1 and sub G1 cell population (%) increased. When 5-FU was added to the cells transfected with LMP2A siRNA, its anti-proliferative effect increased and the expression of p-AKT decreased. In sequential combination of 5-FU and LY, the expression of p-p53 was increased and bcl-2 expression was diminished compared to 5-FU alone.

**Conclusion:**

These data suggest that sequential combination of 5-FU and LY induce synergistic cytotoxicity and overcome intrinsic and acquired resistance of 5-FU via downregulation of activated p-AKT and mitochondria-dependent apoptosis in EBV gastric cancer cell line, SNU-719.

## Background

The worldwide incidence of gastric adenocarcinoma is estimated to exceed 75,000 cases/year, and recent studies have shown that Epstein-Barr virus (EBV) is associated with 10%-18% of gastric cancers. In Korea, EBV-positive cells are found in 7%-10% of gastric cancers and the occurrence of EBV-positive gastric cancers is estimated to be around 4,500-6,400 cases/year based on the fact that gastric cancer has the highest incidence of all cancers.

EBV not only causes infectious mononucleosis, but is also a herpes virus with oncogenic potential, giving rise to Burkitt's lymphoma, nasopharyngeal carcinoma, Hodgkin's disease, B-cell lymphoma in immunodeficient patients, and gastric carcinoma [1].

Of the six types of identified EBV nuclear antigens (EBNAs), only EBNA1 is expressed in gastric carcinoma, and of the three latent membrane proteins (LMPs), LMP1 and LMP2B are not expressed, although LMP2A is expressed in some cases. The *BARF0 *gene in the *Bam*HI-A region and the EBER genes (*EBER1 *and *EBER2*) are always expressed. The transcription of these genes is tightly regulated to maintain the virus in a dormant state in host cells [2].

EBV-based strategies for treating EBV-positive cancers include the prevention of viral oncogene expression, eliminator of the EBV episome, and induction of the EBV infection to the lytic cycle. Ganciclovir (GCV) is an antiviral drug that can be used to treat cancers if the virus in the tumor cells becomes lytic. Host cells with the lytic type of EBV infection, but not the latent type, express virally encoded kinases that can phosphorylate the prodrug, GCV, and convert it to its active cytotoxic form. Furthermore, phosphorylated GCV can be transferred to nearby cancer cells, thus inducing 'by-stander killing.' Because EBV-positive gastric tumor cells are primarily infected with the latent form of EBV, GCV itself is not effective in treating EBV-positive gastric cancers until the virus enters its replicative lytic cycle [3,4].

A recent study confirmed that chemotherapeutic agents (5-fluorouracil [5-FU], cisplatin, and paclitaxel) induce the expression of the immediate early proteins BMRF1, BZLF1, and BRLF1 [4]. Both BZLF1 and BRLF1 are transcription factors that activate the transcription of other genes involved in the lytic conversion of the virus. Three different signal transduction pathways (the p38 stress mitogen-activated protein kinase (MAPK), phosphatidylinositol 3-kinase (PI3K), and protein kinase C δ pathways) are known to be important in the induction of lytic EBV infections by cytotoxic chemotherapeutic agents.

The main cause of treatment failure in advanced gastric cancer is the development of chemoresistance to cytotoxic chemotherapies. The chemoresistance to chemotherapeutic drugs probably involves an anti-apoptosis effect. Therefore, a novel therapeutic strategy is required to target cellular signaling molecules in the presence of the virus in malignant cells.

LMP2A is expressed as a transmembrane protein in latently infected cells at latency stages I, II, and III. This viral protein drives the proliferation and survival of B cells in the absence of signaling through the B-cell receptor and simultaneously activates the PI3K/AKT pathway, which is involved in the survival of latently infected B cells [5]. It has transforming capacity, alters epithelial cell motility, and inhibits epithelial cell differentiation. LMP2A expression influences the expression of a range of genes involved in cell-cycle induction, the inhibition of apoptosis, and the suppression of cell-mediated immunity [6]. In addition to viral proteins, changes in cellular signaling molecules caused by EBV contribute to resistance to the cytotoxic anti-cancer drugs (5-FU, cisplatin, and paclitaxel) used for gastric cancer. A number of cellular mechanisms that contribute to chemoresistance have been described. These include upregulation of the multidrug-resistance gene product and mutation of the p53 tumor suppressor gene, which impairs the p53-dependent induction of apoptosis [7,8]. EBV-positive gastric carcinomas tend to express much higher amounts of p53 than do EBV-negative carcinomas [2,9]. Leung *et al *[10] reported that EBV-positive gastric carcinomas show weak to moderate p53 expression at various stages of the disease, indicating a role for EBV in a non-mutational mechanism of p53 overexpression. High Bcl-2 expression in EBV-positive gastric carcinoma might protect tumor cells from apoptosis [11].

Previous in vitro studies have reported that several chemotherapeutic agents, including 5-FU, paclitaxel, vinblastine, vincristine, daunomycin, and doxorubicin, can activate nuclear factor κB (NF-κB), and that this response results in marked suppression of the cell's apoptotic potential [12,13].

AKT, a serine/threonine kinase, is a key molecule in protecting cells from apoptosis, and the AKT-mediated survival signaling pathway is an attractive target for cancer chemotherapy. The activation of AKT inactivates the expression of caspase 9 and regulates the expression of the apoptosis-inducing FAS ligand [14,15]. It also phosphorylates IκB, promoting IκB degradation, thereby increasing the activity of the well-known cell survival factor, NFκB. The expression of AKT is altered in various human tumors, and this aberrant expression may contribute to chemoresistance [16-18]. AKT-mediated chemoresistance is likely to result from overall anti-apoptotic activity of AKT and activation of the PI3K signaling cascade, which leads to multidrug resistance.

It has been reported that cytotoxic chemotherapeutic agents, including 5-FU, doxorubicin, and cisplatin, can induce lytic EBV gene transcription in latently infected EBV-positive cell lines, and that the EBV viral protein LMP2A activates the PI3K/AKT pathway, which leads to maintenance of the latent form [19]. We examined whether treatment with 5-FU or LY294002 alone or in combination induces the activation of phosphorylated AKT (p-AKT). We also investigated whether the inhibition of p-AKT enhances the growth inhibitory and apoptotic effects of chemotherapeutic agents in gastric cancer cells. The object of this study was to evaluate the role of p-AKT in inducible chemoresistance and to overcome this resistance by 5-FU/LY2940002 combination treatment.

## Methods

### 1) Cell culture and reagents

The EBV-negative AGS gastric cancer cell line and the EBV-positive SNU-719 gastric cancer cell line were obtained from the Korea Cell Line Bank (Seoul, Korea). They were maintained in Roswell Park Memorial Institute (RPMI) 1640 culture medium supplemented with streptomycin (100 g/mL) and penicillin (100 U/mL), glutamine (2 mM), and 10% (v/v) fetal bovine serum (complete medium). The cells were grown at 37°C in a humidified atmosphere containing 5% CO_2_. LY294002 and 5-FU were purchased from Sigma (St Louis, MO). They were dissolved in dimethyl sulfoxide (DMSO) before use in the cytotoxicity assay. The final concentrations of the DMSO were 0.1% or less in drugs. Methanethiosulfonate (MTS) was purchased from Promega (San Luis Obispo, CA).

### 2) Treatment schedules

The cell lines exhibited a wide range of sensitivity to 5-FU and LY294002 in single and sequential treatments. Combination studies involved treatment with an initial 24 h or 48 h exposure to 10 μM 5-FU, followed by additional 24 h or 48 h treatment with 20 μM LY294002.

### 3) Cytotoxicity

AGS and SNU-719 cells were seeded in 96-well plates at pre-determined cell densities (2 × 10^3 ^cell/100 μL/well and 7 × 10^3 ^cell/100 μL/well, respectively). After overnight incubation to allow the cells to attach to the bottoms of the wells, the cells were treated singly with 5-FU (0.1-300 μM) or LY294002 (0.1-80 μM) diluted in DMSO for 24 h, 48 h, or 72 h, or with a sequential application of the drugs.

Cell viability was evaluated with an MTS assay 72 h after exposure to the drugs. The sensitivity of tumor cells to 5-FU (5-FU treatment alone or in combination with LY294002) was determined by estimating the IC_50 _values (doses that induce 50% growth inhibition) for 5-FU from the dose-response curves. Interactions between 5-FU and LY294002 were expressed as the combination index (CI) on a CI-isobologram (Kanazawa et al., 1997): ≤ 0.8 represents synergistic cytotoxicity; 0.8 < CI <1 represents additive cytotoxicity; and ≥ 1 represents antagonistic cytotoxicity.

### 4) Western blotting

AGS and SNU-719 cells were cultured in 100 mm dishes and treated with 5-FU or LY294002 using the protocols described above. The cells were lysed with lysis buffer (0.02 M Tris, 0.15 M NaCl, 0.1% sodium dodecyl sulfate [SDS], 1% Triton X-100, 1% sodium deoxycholate [pH 7.5 with HCl], 0.02 mM phenylmethylsulfonyl fluoride, 0.1 mM NaF, 0.01 mg/mL leupeptin, 0.01 mg/mL pepstatin) and then centrifuged at 12,000 × g for 30 min at 4°C. The amount of protein was determined with the Bradford protein assay (Bio-Rad). The lysates (40 μg/lane) were boiled for 5 min, separated by 10%-12% SDS-polyacrylamide gel electrophoresis, and transferred to polyvinylidene difluoride membrane (Amersham Biosciences, Piscataway, NJ). The membranes were incubated for 1 h with blocking buffer (5% nonfat milk and 0.1% Tween 20 in Tris-buffered saline [TBS-T]) and overnight with mouse monoclonal antibodies directed against basic fibroblast growth factor (1:1000 dilution; Santa Cruz Biotechnology Inc., Santa Cruz, CA), p-NFκB (1:200 dilution; Cell Signaling), p-AKT (1:500 dilution; Santa Cruz Biotechnology Inc.), or total AKT (1:500 dilution; Cell Signaling), or rabbit monoclonal antibodies directed against p-p53, Bcl-2, or Bak1 (1:2,000; Santa Cruz Biotechnology Inc.), or mouse polyclonal antibody directed against BAX (1:2,000; Santa Cruz Biotechnology Inc.). The membranes were washed three times with TBS-T and incubated for 1 h with horseradish-peroxidase-conjugated donkey anti-mouse IgG or donkey anti-rabbit IgG antibody (Santa Cruz Biotechnology Inc.). The proteins were detected with enhanced chemiluminescence reagent (ECL kit, Amersham Biosciences).

### 5) Cell-cycle analysis

Cells were seeded in 150 mm dishes (5-7 × 10^5 ^cells/dish) and treated with 5-FU (10 μM) or LY294002 (20 μM) alone or sequentially in combination for 24 h, 48 h, or 72 h. The cells were harvested and fixed overnight with cold 70% ethanol at -20°C. The fixed cells were washed with phosphate-buffered saline, stained with 0.05 mg/mL propidium iodide, and treated with 1 mg/mL RNase A in a water bath (37°C) for 15 min. Analyses of 10,000 events were acquired on a FACS Calibur flow cytometer (Becton Dickinson Biosciences), and the cell cycles were analyzed using the ModFit DNA analysis software (Verity Software House).

### 6) Apoptosis

Apoptosis was measured by flow cytometry after concurrent staining with fluorescein-conjugated annexin V and propidium iodide (Annexin V-FITC kit, Becton Dickinson). In brief, AGS and SNU-719 cells were treated with 5-FU for 48 h, then with LY294002 for 24 h, and the cells were collected, washed, and stained with annexin V-propidium iodide before being subjected to flow-cytometric analysis.

In another experiment, the nuclear chromatin of the cells was stained with the fluorogenic compound 4', 6-diamidino-2-phenylindole (DAPI) to assess any morphological changes. SNU-719 cells (about 1 × 10^5 ^cells) were treated with 5-FU or LY294002 singly or in combination, as described previously, then fixed in 10% formaldehyde before the cells were attached to glass microscope slides by Cytospin at 700 rpm for 5 min. The slides were then stained with DAPI solution (1:500, Pierce) for 10 min. The cells were visualized under a fluorescence microscope with a blue filter (330-380 nm) to identify any morphological features of apoptosis, such as cell shrinkage, chromatin condensation, and the formation of apoptotic bodies (AX70, Olympus). All the experiments were performed in triplicate.

### 7) RNA interference

RNA duplexes were synthesized by Samchullypharm (Korea). The siRNA target sequence for LMP2A mRNA was 5'-AACUCCCAAUAUCCAUCUGCU-3'. The LMP2A siRNA was designed such that it did not overlap sequences shared by LMP2B. A control scrambled siRNA duplex was also produced by Samchullypharm (Scramble II Duplex). The siRNA duplex was transfected using Lipofectamine2000 Reagent (Invitrogen) as recommended by the manufacturer and the cells were assayed for silencing 2 days after transfection. All experiments were performed in triplicate, and at least two independent experiments were performed for each of the cell lines.

### 8) Statistical analysis

All data are expressed as the mean ± standard deviation (SD) of at least three independent experiments. Two-tailed, paired Student's *t *test and one-way ANOVA were used to determine the differences between the control and treatment groups (Analyze-It software for Microsoft Excel).

## Results

### 1) Enhanced cytotoxic effect of 5-FU with the PI3K inhibitor LY294002

**Figure 1:**
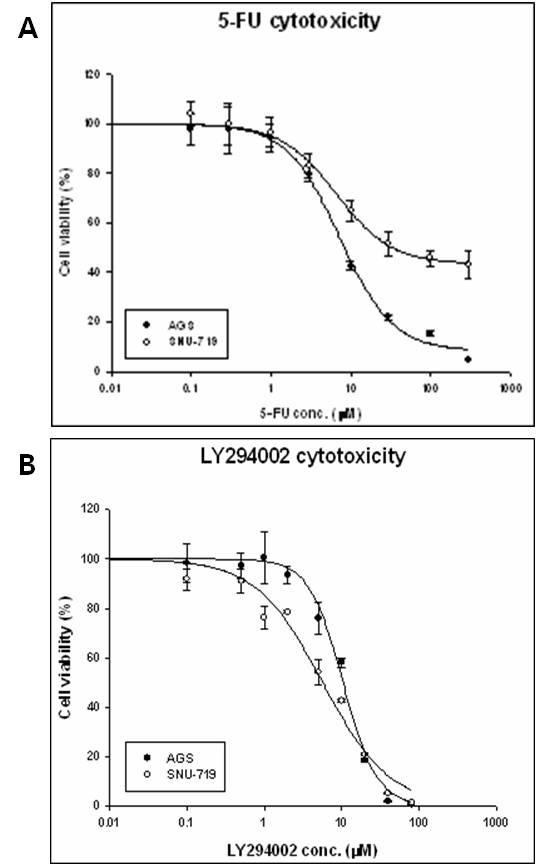
**Dose-response curves for single 5-FU and LY294002 treatments of AGS and SNU-719 gastric cancer cell lines**. Dose-dependent inhibition of cell proliferation by 5-FU and LY294002 in gastric cancer cells. Cells were treated with increasing concentrations of 5-FU (A) or LY294002 (B) for 72 h, and the viable cells were quantified by MTS assay. The data presented are the mean ± SD of three independent experiments.

**Figure 2:**
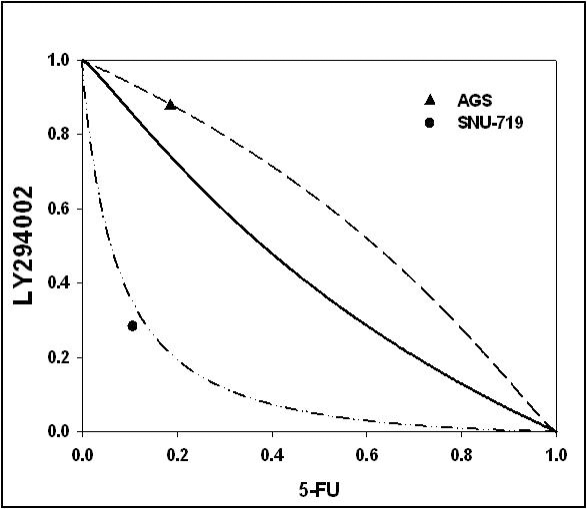
**Isobolograms showing the interaction between 5-FU and LY294002 in AGS and SNU-719 cells**. Synergistic analysis of the interaction between 5-FU and LY294002 in AGS and SNU-719 gastric cancer cells. The interaction between 5-FU and LY294002 given on the schedule 5-FU → LY294002 is shown with an isobologram technique. The experimental isoeffect points are the concentrations (expressed relative to the 5-FU and LY294002 IC_50 _concentrations) of the two agents which, when combined, reduced cell viability by 50%. The data points above the diagonal line of the additive effects in the isobole suggest antagonism; those below the diagonal line suggest synergism. The isobologram analysis was based only on data obtained directly from actual experiments.

5-FU or LY294002 was applied to SNU-719 and AGS cells at different drug concentrations (0.1-300 μM) for 72 h. The cytotoxicity of 5-FU was measured in EBV-negative AGS cells and EBV-positive SNU-719 cells, which produced IC_50 _values of 11.6 ± 9.2 μM and 22.9 ± 2.8 μM (*P *< 0.01), respectively. IC_50 _for 5-FU in SNU-719 cells was two-fold higher than that in AGS cells, with approximately 30% of cells remaining alive at concentrations of more than 300 μM (Figure 1A). In contrast, the IC_50 _values for LY294002 in SNU-719 and AGS cells were not significantly different, being 5.1 ± 2.4 μM and 8.9 ± 0.6 μM (*P *> 0.05), respectably (Figure 1B). We hypothesized that if the induction of the PI3K/AKT pathway by LMP2A contributes to 5-FU resistance in EBV-positive gastric cancer cells, the combination of 5-FU with specific inhibitors of the PI3K pathway may result in a synergistic effect in EBV-positive gastric cancer cell lines, such as SNU-719 cells. Combined treatments with various concentrations of 5-FU and LY294002 resulted in the reduced growth of SNU-719 cells compared with that observed with 5-FU treatments. The CI values for 5-FU and LY294002 were calculated from the results of the experiments presented in Figure 2. When the isobologram was analyzed, the CI values were 0.36 and 1.1 in EBV-positive (SNU-719 cells) and EBV-negative (AGS cells) gastric cancer cells, respectively. This indicates that 5-FU and LY294002 used in combination act with synergistic effect in SNU-719 cells and additive effect in AGS cells (Figure 2).

### 2) The combination of 5-FU and LY294002 influences the expression of downstream signal molecules

To verify that the antiproliferative effect of 5-FU combined with LY294002 is attributable to the inhibition of the PI3K and/or NFκB signal pathways, we investigated the activation status of their downstream components by Western blot analysis.

**Figure 3:**
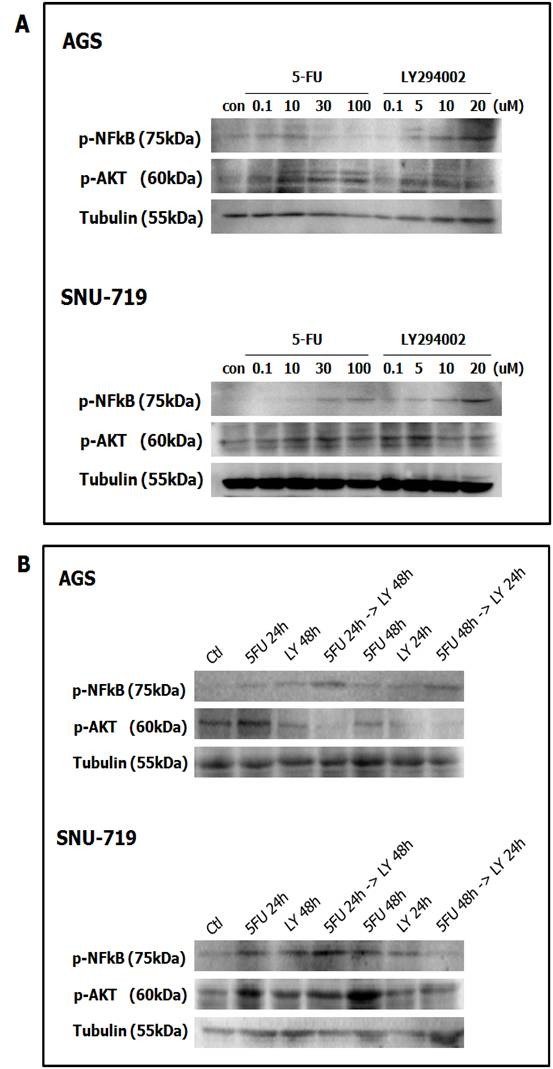
**Expression of phosphorylated AKT and phosphorylated NFκB in AGS and SNU-719 gastric cancer cells measured by Western blot analysis**. (A) Dose-dependent expression of p-AKT and p-NFκB in AGS and SNU-719 gastric cancer cells. (B) Schedule-dependent expression of p-AKT and p-NFκB in AGS and SNU-719 gastric cancer cells. The data are the mean ± SD of three independent experiments.

5-FU induced the expression of p-AKT in a dose-dependent manner in both SNU-719 and AGS cells. 5-FU treatment also increased the expression of phosphorylated NFκB (p-NFκB) in SNU-719 cells, but reduced it in AGS cells. In contrast, LY294002 reduced p-AKT expression but increased p-NFκB expression in a dose-dependent manner (Figure 3A). In AGS cells, two sequential treatments with 5-FU (at 24 h and 48 h) followed by LY294002 (at 48 h and 24 h) reduced p-AKT and increased p-NFκB expression to a greater degree than did 5-FU alone (24 h). The changes in p-AKT and p-NFκB expression in SNU-719 cells were similar to those in AGS cells when 5-FU was combined with LY294002 in a sequential manner (5-FU at 24 h → LY294002 at 48 h). However, sequential treatment with 5-FU (48 h) followed by LY294002 (24 h) markedly reduced the expression of both p-AKT and p-NFκB compared with that in cells treated with 5-FU alone for 24 h or 48 h (Figure 3B).

In SNU-719 EBV-positive gastric cancer cells, the basal expression of p-AKT may be enhanced through the LMP2A-mediated amplification of the PI3K/AKT pathway, conferring resistancy to 5-FU treatment. Thereby, we examined if chemoresistance to 5-FU was caused by the induction of p-AKT expression as well as p-NFκB expression. Our data suggest that the reduced expression of p-AKT and p-NFκB following LY294002 treatment overcomes 5-FU resistance in EBV-positive gastric cancer cells.

### 3) Combination of 5-FU and LY294002 affects the cell cycle and its regulators

**Figure 4:**
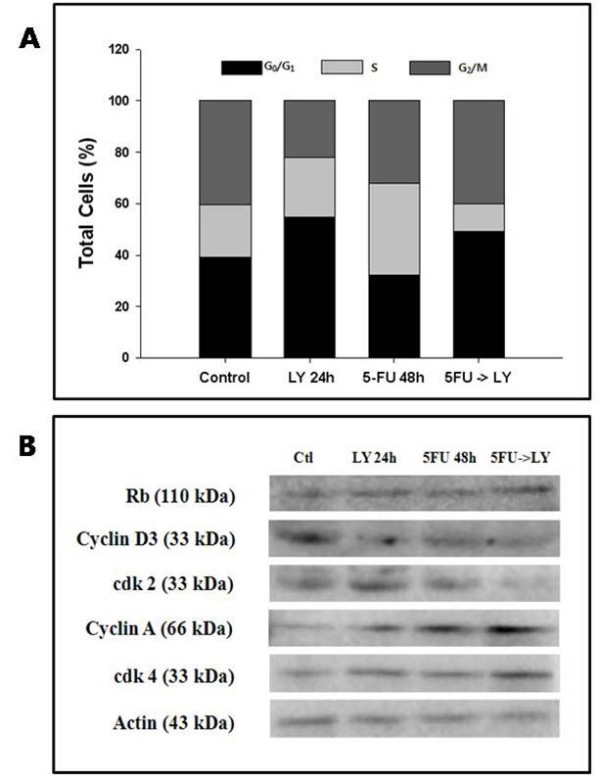
**Cell-cycle effects of 5-FU and LY294002**. SNU-719 cells were incubated with 5-FU (10 μM) or LY294002 (20 μM), in single or combined treatments, for 72 h. (A) Cell-cycle distributions were measured by flow cytometry. The results are expressed as the mean percentages of cells in the different phases of the cell cycle in each sample (sum to 100%) in three independent experiments. (B) Western blot analysis of retinoblastoma 1 (RB1), cyclin D3, CDK2, cyclin A, and CDK4 was performed using the corresponding antibodies to confirm the phase-specific cell-cycle effects of 5-FU and LY294002 alone or in combination, as shown in (A). Representative immunoblots are shown from three independent experiments. Actin on the same immunoblot was used as the loading control.

The cell-cycle distribution was analyzed in SNU-719 cells exposed to 5-FU or LY294002 singly or in combination for 72 h by flow cytometry and Western blotting. 5-FU (48 h) induced S-phase arrest in 32.5 ± 1.5% of the total cell population and LY294002 (24 h) induced G_0_/G_1 _phase arrest in 54.8 ± 3.5% of cells. Treatment with 5-FU (48 h) followed by treatment with LY294002 (24 h) induced G_0_/G_1 _phase arrest in 49.3 ± 7.5% of cells, S phase in 10.8 ± 5.9% of cells, and G_2_/M phase in 39.9 ± 4.3% of cells (Figure 4A).

The expression of phase-specific cyclins and cyclin-dependent kinases (CDKs), as determined by immunoblotting in parallel experiments, was compatible with the cell-cycle distribution (Figure 4B). 5-FU increased the expression of cyclin A (Figure 4B, lane 3) compared with that in untreated cells (Figure 4B, lane 1), which is consistent with S-phase arrest. LY294002 inhibited the expression of cyclin D3 and slightly increased the expression of CDK2, CDK4, and cyclin A (Figure 4B, lane 2) compared with that in untreated cells (Figure 4B, lane 1), consistent with G_1_-phase arrest. Compared with 5-FU treatment (48 h), combined treatment with 5-FU and LY294002 downregulated the expression of cyclin D3 and CDK2, and upregulated the expression of cyclin A and CDK4 (Figure 4B, lane 4).

### 4) Sequential combination of 5-FU with LY294002 leads to increased apoptotic cell death

**Figure 5:**
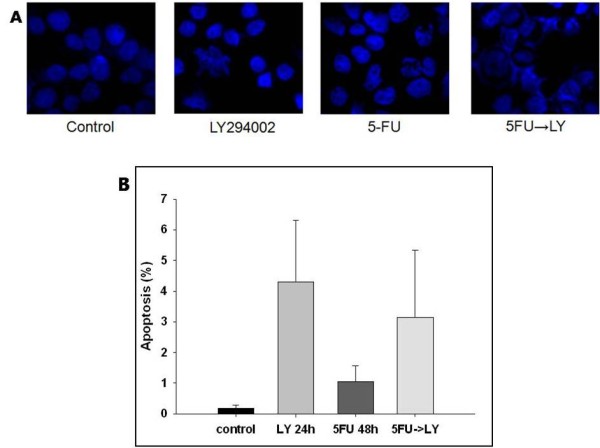
**Apoptotic cell death induced by 5-FU, LY294002, or their combination**. (A) Morphological changes were induced in SNU-719 cells by combined treatment with 5-FU and LY294002. Nuclear fragmentation and condensation were evident in the cells treated with the sequential combination of 5-FU and LY294002. (B) Flow-cytometric analysis of apoptosis was performed. The untreated cells and cells treated with the experimental drugs were stained with propidium iodide and annexin V. The values are the averages of three independent experiments (mean ± SD).

To investigate whether the sequential combination of drugs induces apoptotic cell death more effectively than does the individual treatments, we looked for morphological changes and quantitatively measured the apoptotic cell population in SNU-719 cells treated with 5-FU, LY2940002, or their sequential combination. The cells treated with the sequential combination (5-FU followed by LY294002) showed nuclear fragmentation with DAPI staining (Figure 5A). We observed DNA condensation and large nuclei in the cells treated with 5-FU (48 h) follow by LY294002 (24 h). Sequential treatment with 5-FU and LY294002 resulted in a mixed pattern of the morphological changes observed in the cells treated with each drug individually. The apoptotic cell populations were also measured by flow cytometry to analyze their cellular DNA contents after staining with propidium iodide and annexin V (Figure 5B). After the combination treatment, apoptotic cells increased to a greater extent than in cells treated with 5-FU alone.

### 5) Knockdown of LMP2A enhances the antiproliferation effect of 5-FU treatment

**Figure 6:**
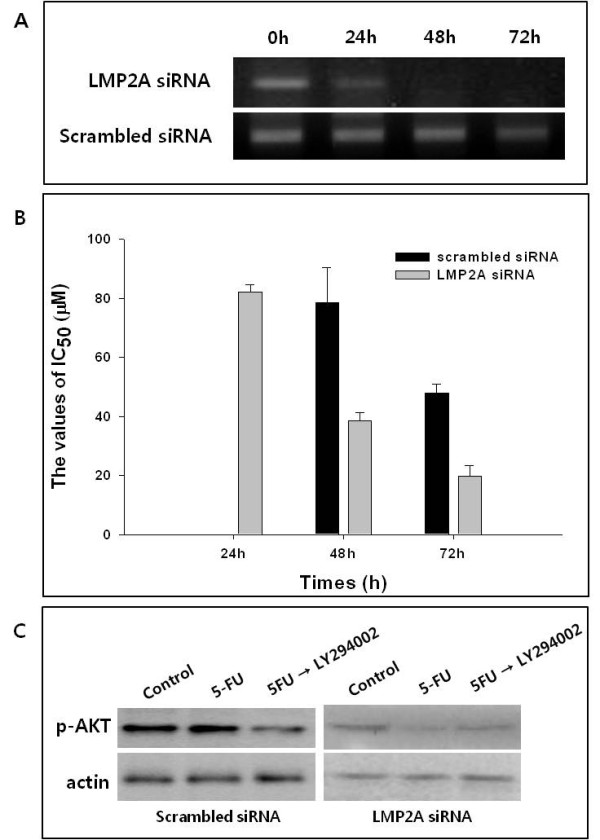
**Cytotoxic effects of 5-FU and the expression of p-AKT by drug treatment in cells transfected with LMP2A siRNA**. (A) LMP2A siRNA-silenced SNU-719 cells were subjected to RT-PCR for LMP2A in a time-dependent manner. (B) The inhibitory effects on cellular proliferation caused by 5-FU treatment were more pronounced in SNU-719 cells transfected with LMP2A siRNA than in cells transfected with the scrambled siRNA. (C) The expression of p-AKT was reduced further by 5-FU in cells transfected with LMP2A siRNA compared with in cells transfected with the scrambled siRNA (NC).

To assess whether the chemoresistance to 5-FU in SNU-719 cells is attributable to LMP2A, we knocked down LMP2A mRNA transcripts using LMP2A siRNA, and compared the antiproliferative effects of 5-FU treatment in a time-dependent manner. LMP2A siRNA caused over 90% of reduction in LMP2A mRNA expression compared to scrambled siRNA (Figure 6A). Because the survival cell population (%) still was over 50% despite of high concentration of 5-FU (< 1 mM), the IC_50 _of 5-FU treatment for 24 h could not be calculated after transfected with the scrambled siRNA, but the IC_50 _of 5-FU was 82.2 ± 2.5 μM in cells transfected with LMP2A siRNA. IC_50 _of the 5-FU treatment for 48 h for LMP2A-siRNA-transfected SNU-719 cells (36.0 ± 2.7 μM) was two-fold lower than that for cells transfected with scrambled siRNA (78.6 ± 11.6 μM). Similar results were observed following treatment with 5-FU for 72 h (Figure 6B). When cells transfected with LMP2A siRNA were treated singly with 5-FU or with the combined drugs (5-FU followed by LY294002), the expression of p-AKT was significantly reduced than that in cells transfected with the scrambled siRNA (Figure 6C).

### 6) Combination of 5-FU with LY294002 induces synergistic cytotoxicity via activation of the mitochondria-dependent apoptotic pathway

**Figure 7:**
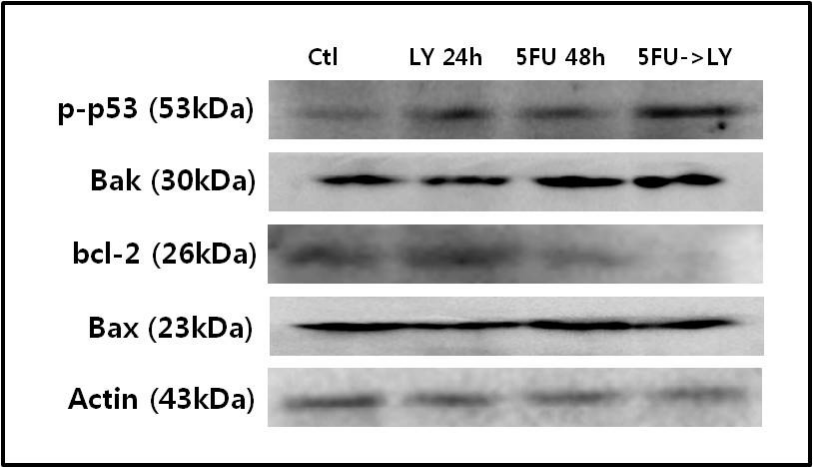
**Western blot analysis of Bcl-2 family members (Bak1, Bcl-2, and Bax) and p-p53**. The expression of Bak1, Bax, Bcl-2, and β-actin proteins in SNU-719 cells was investigated. Sequential treatment with 5-FU followed by LY2940002 significantly reduced the expression of Bcl-2 protein but slightly increased the expression of Bak1. However, there was no change in the expression of Bax protein.

We evaluated changes in the signaling proteins of the mitochondria-dependent apoptotic pathway, which are known to be constitutively expressed in EBV-infected cancers, when 5-FU was combined with LY294002. Bcl-2 is a protein of the anti-apoptotic family, and Bak1 and Bax are proteins of the pro-apoptotic family. Single treatment with LY294002 did not change the expression of Bcl-2 or Bak1 proteins compared with that in the control. However, the expression of Bcl-2 was reduced and that of Bak1 was slightly increased in cells treated singly with 5-FU. When the two drugs were combined sequentially, the expression of Bcl-2 was completely diminished and the expression of Bak1 was similar to that after a single treatment with 5-FU. There was no change in Bax expression after any treatment. When the two drugs were applied separately to the cells, p-p53 was slightly increased relative to that in the control. Its expression was more significantly increased after the sequential combination treatment than after individual treatment with either of the two drugs (Figure 7).

## Discussion

EBV-positive gastric cancer cell lines have been identified by several investigators, by detecting EBV nuclear antigens (EBNAs), latent membrane protein 1 (LMP1), or BARF0 [2,20]. However, the frequency of LMP2A expression is lower in patients with EBV-positive gastric cancer and in EBV-positive gastric cancer cell lines than in B-cell malignant lymphomas [21-23]. It is difficult to treat EBV-positive gastric cancer successfully because significant resistance to cytotoxic anti-cancer drugs, such as paclitaxel, cisplatin, and 5-fluorouracil (5-FU), has been demonstrated in laboratory studies and clinical trials [24,25]. Only the lytic form of EBV is affected when infected cancer cells are treated with gancyclovir (GCV), and antiviral drugs are ineffective in some gastric cancer cells, such as SNU-719, because EBV lytic genes are expressed at very low levels [19]. Recently, various experimental treatments for EBV-positive gastric cancers have been studied to improve their efficacy. Representative approaches include the combination of 5-aza-2-deoxycytidine or histone deacetylase (HDAC) inhibitors with cytotoxic anti-cancer drugs or with antiviral drugs such as GCV [26,27]. We consider that epigenetic modification by HDAC is not essential for changing the latent form of EBV into the lytic form, because it does not directly affect cellular signal transduction pathways.

If the MAPK signal pathway or the phosphatidylinositol-3-kinase (PI3K)/AKT signal pathway is dysregulated in epithelial cells or B-cell lymphomas, EBV is likely to be changed into the lytic form [19,20]. Therefore, we hypothesized that such cellular signal transduction pathways play important roles in the transformation of EBV into the lytic form, and that resistance to cytotoxic anti-cancer drugs can be overcome if these pathways are effectively inhibited. We also assumed that LMP2A has a greater effect on cellular signal transduction pathways, in terms of converting EBV into the lytic form, than do other viral proteins. Our results confirmed that EBV-positive gastric cancer cells are resistant to 5-FU and that this resistance is reduced when the expression of phosphorylated nuclear factor kappaB (p-NFκB) and phosphorylated AKT (p-AKT) is decreased by combining 5-FU with LY294002, a PI3K inhibitor (Figure 3). Furthermore, apoptosis was enhanced not only when the PI3K/AKT signal pathway is inhibited, but also when p-p53 expression is increased and Bcl-2 expression is reduced (Figure 7).

Even at high concentrations of 5-FU, the number of drug-resistant SNU-719 cells was 20-30% higher than that for the AGS EBV-negative gastric cancer cells. In contrast, LY294002 strongly affected both SNU-719 cells and AGS cells, regardless of their EBV status. However, when EBV-positive gastric cancer cells were treated with enzastaurin, which inhibits both PI3K and protein kinase C (PKC), no cytotoxic effects were observed (data not shown). Lee et al. [28] reported that synergistic or additive effects were observed in diverse gastric cancer cell lines when treated with enzastaurin alone or in combination with cytotoxic anti-cancer agents; however, significant resistance to enzastaurin treatment alone was observed only in SNU-719 cells (IC_50 _> 250 μM).

Among the various reported mechanisms of resistance to 5-FU, such as multi-drug resistant proteins (MRP, MDR1) and DPD, we focused on cell signaling proteins [29]. However, previous *in vitro *studies have reported that several chemotherapeutic agents (e.g., 5-FU, paclitaxel, cisplatin, irinotecan, and doxorubicin) can activate NF-κB, which results in notable suppression of the apoptotic potential [13]. AKT is an important regulator of cell survival and apoptosis. Constitutive activation of NF-κB is observed in various malignant cells, which implies that activated NF-κB induced by AKT may play a major role in the chemo-resistance of gastric cancer cells [16-18,30,31]. The expression of this protein is altered in various human tumors, and this aberrant expression may contribute to cellular chemo-resistance. It phosphorylates IκB, thus promoting IκB degradation and thereby increasing the activity of the well-known cell survival factor, NF-κB.

We investigated how the expression of p-AKT and p-NF-κB changes with the induction of resistance to 5-FU, based on the finding that LMP2A activates the PI3K/AKT signaling pathway [32]. When SNU-719 cells, with the PI3K/AKT signaling pathway activated by LMP2A, were treated with 5-FU, p-AKT expression was increased. LY294002 showed a significant anti-proliferative effect in both SNU-719 and AGS cells, regardless of their EBV status. In particular, it reduced p-AKT expression remarkably in SNU-719 cells and produced excellent cytotoxicity compared with treatment by 5-FU alone (Figure 3). Generally, over-expression of p-NFκB protein is important for drug resistance to 5-FU, but the activation of PI3K/AKT signal transduction induced by LMP2A appears to be another important cause of resistance to 5-FU in EBV-positive gastric cancer cells.

When SNU-719 cells expressing LMP2A were transfected with LMP2A siRNA, the expression of p-AKT was reduced and the anti-proliferative effect of 5-FU was recovered. Despite the high concentration of 5-FU, SNU-719 cells transfected with scrambled siRNA survived, but LMP2A-knockdown SNU-719 cells were significantly reduced (Figure 6). The changes in gene or protein expression that facilitate the development of gastric cancer have yet to be identified. However, dysregulation of the PI3K/AKT signal pathway after EBV infection is a possible mechanism in the carcinogenesis of gastric cancer.

LY294002 was combined with 5-FU for different sequential treatments and exposure times. When LY294002 was followed by 5-FU, the expression of p-AKT was increased and an antagonistic effect was observed as a combination index [CI] value of 1.1 (data not shown). However, the reverse sequential combination (*i.e*., 5-FU followed by LY294002) significantly reduced p-AKT expression with a highly synergistic effect (CI = 0.36). These results indicate that p-AKT contributes to the resistance of EBV-positive gastric cancer cells to 5-FU.

The expression of p-NFκB decreased in AGS EBV-negative gastric cancer cells, depending on the concentration of 5-FU when used alone. However, it increased when used in combination with LY294002. The CI value obtained for the two drugs indicated additive effects. In contrast, p-AKT expression increased after treatment with 5-FU alone, but decreased when 5-FU was combined with LY294002. In AGS EBV-negative gastric cancer cells, it is considered that apoptosis is induced by inhibition of the NF-κB signal pathway in the case of treatment with 5-FU alone, and predominantly by the inhibition of p-AKT expression and its downstream signaling molecules in the case of treatment with LY294002.

We observed that 5-FU increased the expression of cyclin A, resulting in S-phase arrest of the SNU-719 cell population. Compared with 5-FU alone, the combination of 5-FU with LY294002 downregulated the expression of cyclin D3 and CDK2, and upregulated the expression of cyclin A and CDK4. Sequential treatment with 5-FU followed by LY294002 resulted in a mixed pattern of DNA condensation and large nucleated cells, when compared with cells treated with the individual drugs. When SNU-719 cells are treated with 5-FU or LY294002, alone or in combination, it is postulated that apoptosis is induced by the inhibition of DNA synthesis or G_0_/G_1 _arrest *via *increased p-p53 expression and reduced p-NF-κB expression. It was also confirmed that apoptotic cells were more significantly augmented by increased p-p53 and decreased Bcl-2 expression using combined treatment than using 5-FU treatment alone. Leung et al. [10] reported that most EBV-positive gastric cancer cells express p53 protein at low to medium levels and that another mechanism exists which induces the over-expression of p53, in addition to the direct mutation of p53 by EBV.

It is considered that the high levels of Bcl-2 expression adopted as protection from apoptosis in EBV-positive gastric cancer cells result in the natural death of fewer cancer cells than in the case of EBV-negative gastric cancer cells. In addition, increased Bcl-2 expression results in chemo-resistance to anti-cancer drugs by inhibiting p53-mediated apoptosis [11,33]. However, some studies have reported the absence of altered Bcl-2 expression or p53 accumulation in EBV-positive gastric cancers [33]. Additional research is warranted regarding the roles of Bcl-2 and p53 in the development of drug resistance [34]. In EBV-positive SNU-719 cells, LY294002 enhanced the sensitivity to 5-FU by downregulating activated p-AKT and its downstream molecules, and induced apoptosis by arresting the cell population at G_0_/G_1 _phase. Furthermore, increased sensitivity to 5-FU, with remarkable inhibition of the activated PI3K/AKT signaling pathway, was observed in SNU-719 cells transfected with LMP2A siRNA.

## Conclusions

We demonstrated that the resistance to 5-FU in EBV-positive gastric cancer cells, in which the PI3K/AKT pathway had been activated, was caused by the induction of p-AKT expression as well as p-NF-κB expression. We suggest that the effective inhibition of activated p-AKT by sequential treatment with 5-FU and LY294002 could overcome the 5-FU resistance through increases in anti-proliferation, cell cycle arrest, and induction of mitochondria-independent/-dependent apoptotic pathways.

Progress has been made in overcoming cellular resistance to cytotoxic anti-cancer drugs used for EBV-positive gastric cancers. However, there are several limitations to the treatment strategy of directly eradicating EBV. Further studies are necessary to further improve the therapeutic efficacy by adding antiviral drugs to the combination of 5-FU and PI3K inhibitors, and to establish a standard treatment for patients with EBV-positive gastric cancer.

## Competing interests

The authors declare that they have no competing interests.

## Authors' contributions

JYS carried out the studies and data analyses; JOK participated in the cell culture experiments; SKL, HSC participated in the data analyses and coordination of the study; JHK conceived of the study, and participated in designing study and coordination and helped to draft the manuscript. All authors read and approved the final manuscript.

## Pre-publication history

The pre-publication history for this paper can be accessed here:

http://www.biomedcentral.com/1471-2407/10/425/prepub
